# Two new species of the genus *Gonioctena* Chevrolat, 1836 (Coleoptera, Chrysomelidae) from China

**DOI:** 10.3897/BDJ.12.e132778

**Published:** 2024-09-19

**Authors:** Hee-Wook Cho

**Affiliations:** 1 Nakdonggang National Institute of Biological Resources, Sangju, Republic of Korea Nakdonggang National Institute of Biological Resources Sangju Republic of Korea

**Keywords:** leaf beetle, Chrysomelinae, taxonomy, new species, Fujian, Sichuan

## Abstract

**Background:**

Approximately 120 species of the chrysomelid genus *Gonioctena* are currently known from Palaearctic, Nearctic and Oriental Regions, amongst them, 51 occur in China.

**New information:**

Two new species of the genus *Gonioctena*, *G.klapperichi* sp. nov. (Fujian) and *G.oberthueri* sp. nov. (Sichuan), are described and illustrated from China. Distinguishing characters from closely-related species are presented, respectively. The *Gonioctena* fauna of China now includes 53 species, of which about 60% are endemic. Ovoviviparity is newly recorded in *G.oberthueri* sp. nov.

## Introduction

*Gonioctena* Chevrolat, 1836 is one of the most diverse genera of the subfamily Chrysomelinae, with approximately 120 species in nine subgenera occurring in Holarctic and Oriental Regions (Cho 2023). The main diagnostic features that distinguish *Gonioctena* from other genera are: mid- and hind tibiae each with an angular subapical projection; tarsal claws appendiculate; elytral epipleura visible in lateral view; hind wings well-developed; and procoxal cavities open posteriorly. A large number of *Gonioctena* species exhibit high morphological similarity and considerable colour variation. Therefore, regional reviews and keys of this group generally depend on the shape of male genitalia, for example, Japan (Takizawa 2007), Palaearctic Region (Warchałowski 2010) and China (Yang et al. 2014).

According to the recent catalogue of Palaearctic Coleoptera (Bezděk and Sekerka 2024), China has exceptionally high species richness of the genus *Gonioctena*. There are 51 species of *Gonioctena* in China, accounting for about 40% of world's total, 60% of which are species endemic to China. Amongst them, 12 species have been newly described from China in the past decade. During the examination of materials in the old collections, I found two new species of the genus *Gonioctena* from Sichuan and Fujian Provinces, which are described below. Photographs of dorsal habitus and illustrations of male genitalia are provided. An identification key to Chinese species of *Gonioctena* is not provided in the present study due to the unavailability of some collections for study.

## Materials and methods

The type series of the new species are deposited in the Museum national d’Histoire naturelle, Paris, France (MNHN) and Lev N. Medvedev’s private collection, Moscow, Russia (LMC). Specimens were examined with a Nikon SMZ800 microscope. Male and female genitalia were dissected from adult specimens, softened in a closed Petri dish with wet tissue paper for 6–12 h, cleaned in 10% (w/v) sodium hydroxide and rinsed in distilled water. Photographs were taken using a Nikon D5200 digital camera attached to a Nikon SMZ1500 microscope and were edited in Helicon Focus 5.3.12 and Adobe Photoshop CS5. Line drawings were made from the Photoshop photographs using a Wacom Intuos graphics tablet.

## Taxon treatments

### Gonioctena (Brachyphytodecta) klapperichi sp. nov.

73F277F8-1E6A-5C15-A15C-575775D28A4A

urn:lsid:zoobank.org:act:53783A45-5E76-4EFB-B0ED-48E475E0DCE1

#### Materials

- **Type status:** Holotype. **Occurrence:** recordedBy: Johann Friedrich Klapperich; individualCount: 1; sex: male; lifeStage: adult; occurrenceID: 1983B4D1-CD7A-53CE-8B06-C1C0E9322F90; **Taxon:** scientificName: Gonioctenaklapperichi; kingdom: Animalia; phylum: Arthropoda; class: Insecta; order: Coleoptera; family: Chrysomelidae; genus: Gonioctena; subgenus: Brachyphytodecta; specificEpithet: klapperichi; **Location:** higherGeography: China; stateProvince: Fujian; county: Wuyishan; locality: Guadun; verbatimElevation: 2,300 m; **Event:** eventDate: 08-06-1938; **Record Level:** basisOfRecord: PreservedSpecimen

#### Description

Measurements in mm (n = 1): length of body: 5.30; width of body: 3.60; height of body: 2.40; width of head: 1.57; interocular distance: 1.05; width of apex of pronotum: 1.77; width of base of pronotum: 3.10; length of pronotum along mid-line: 1.40; length of elytra along suture: 4.10.

**Figure 1.**
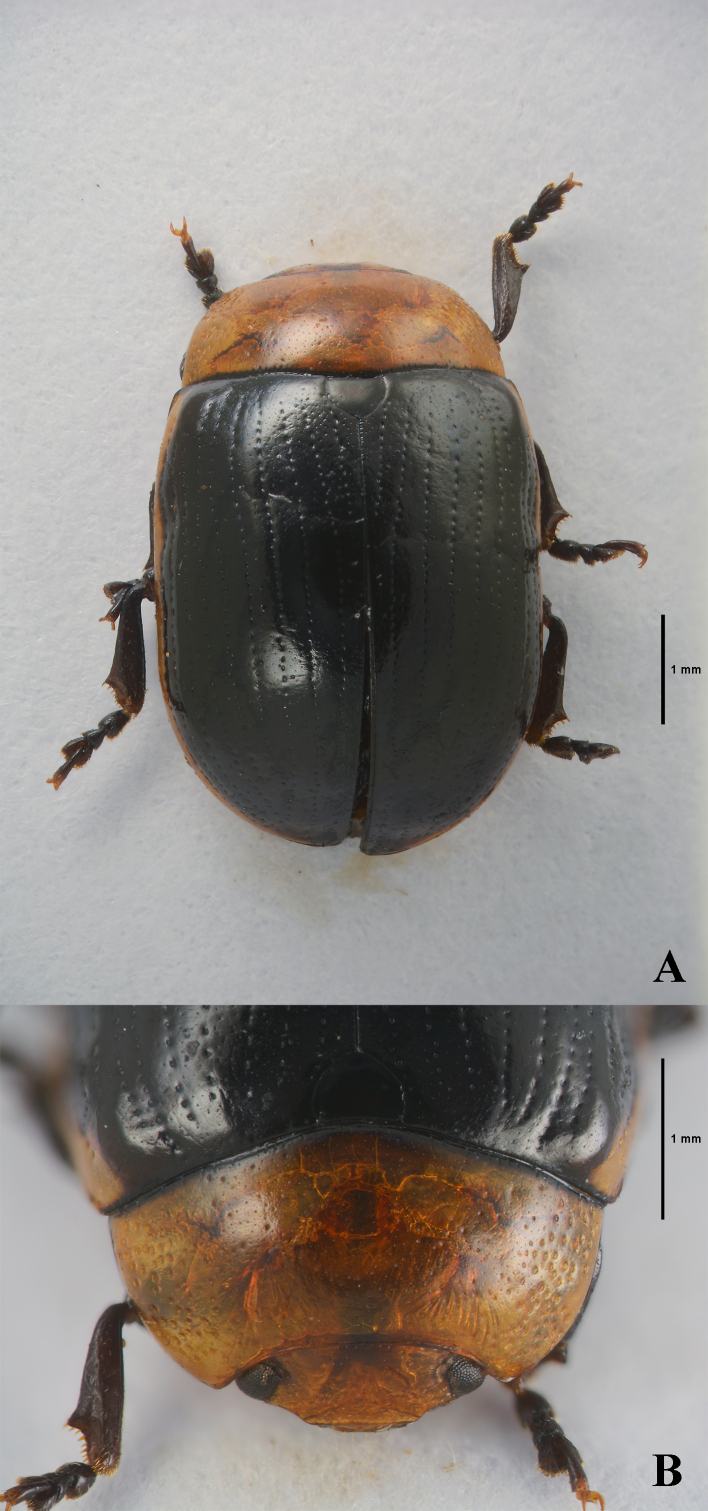
*Gonioctenaklapperichi* sp. nov., holotype. **A** Habitus, dorsal view; **B** Head and pronotum, dorsal view.

Body (Fig. 1) oval and strongly convex. Head yellowish-brown, with an obscure spot on vertex. Mandibles reddish-brown, apex blackish-brown. Maxillary palps blackish-brown. Antennomeres I–II yellowish-brown, III–XI lost. Pronotum yellowish-brown, basal margin black. Scutellum black. Elytra black, lateral margins beyond 10^th^ striae yellowish-brown. Venter blackish-brown, with hypomera and apical margin of last abdominal ventrite yellowish-brown. Legs blackish-brown to black.

Head. Vertex weakly convex, covered with sparse punctures, becoming coarser and denser towards sides. Frontal suture V-shaped, reaching anterior margin, coronal suture rather long, weak. Frons flat, strongly depressed at anterior margin, covered with dense punctures. Clypeus very narrow and trapezoidal. Anterior margin of labrum weakly concave. Mandibles with two sharp apical teeth and large excavation for apical maxillary palpomere on outer side. Maxillary palps four-segmented, with apical palpomere slightly widened, truncate apically.

Pronotum. Lateral sides widest at base, roundly strongly narrowed anteriorly, anterior angles strongly produced. Anterior and lateral margins bordered, lateral margins barely visible in dorsal view. Trichobothria absent on both anterior and posterior angles. Disc covered with sparse or moderately dense punctures; lateral sides covered with much larger and denser punctures; interspaces covered with fine and sparse punctures. Scutellum slightly wider than long, narrowed posteriorly.

Elytra. Lateral sides very slightly widened posteriorly, widest near middle, thence roundly narrowed posteriorly. Humeral calli well developed. Disc covered with eleven regular rows of large punctures, including a short scutellar row; interspaces covered with fine and sparse punctures. Epipleura visible, except near base in lateral view. Hind wings well developed.

Venter. Hypomera weakly rugose, with few punctures near anterolateral corners of prosternum. Prosternum covered with coarse and dense punctures bearing long setae; prosternal process enlarged apically, bordered laterally, with sparse punctures. Metaventrite covered with small and sparse punctures in median region, large and dense punctures in lateral region. Abdominal ventrites covered with sparse or dense punctures bearing short setae.

Legs. Moderately robust. Tibiae widened apically, with tooth-like projection. Fore legs with tarsomere I very lightly narrower than III. Tarsal claws appendiculate.

**Figure 2.**
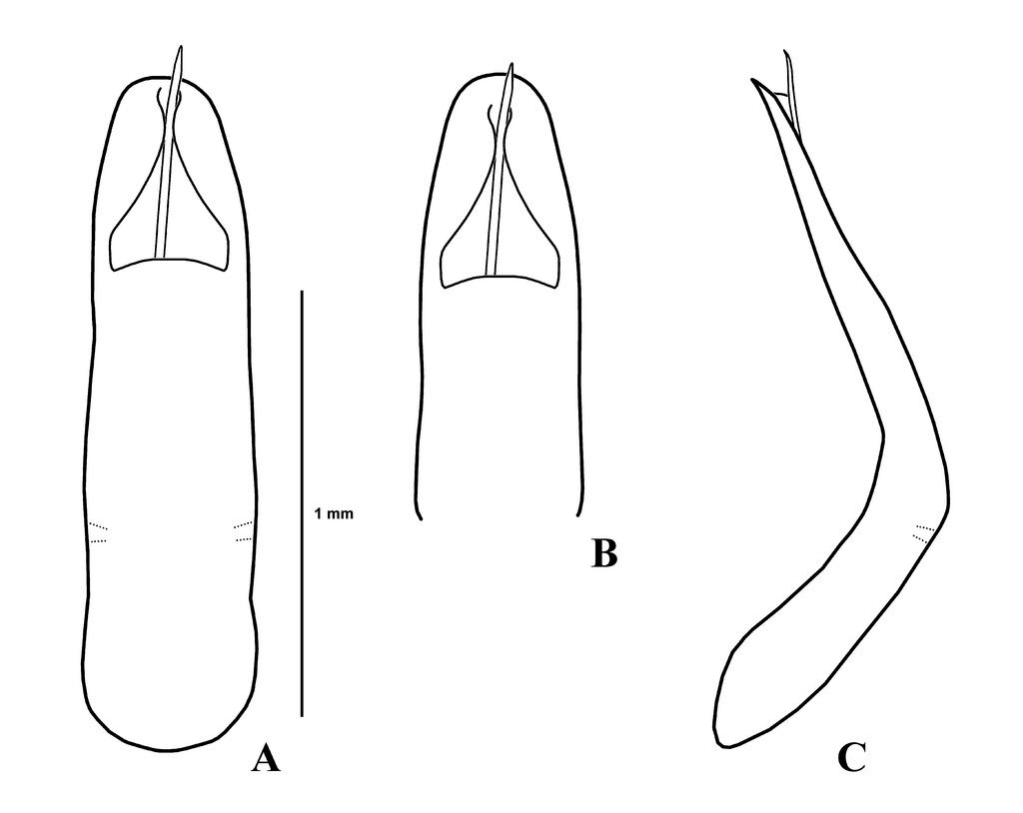
*Gonioctenaklapperichi* sp. nov. **A** Male genitalia, dorsal view; **B** Ditto, apico-dorsal view; **C** Ditto, lateral view.

Genitalia (Fig. 2). Aedeagus rather thick, subparallel-sided, moderately narrowed from apical 1/5 to widely rounded apex in dorsal view; strongly curved, apex pointed in lateral view.

Female: unknown.

#### Diagnosis

*Gonioctenaklapperichi* sp. nov. is similar to *G.scutellaris* Baly, 1862 and *G.melanota* Kippenberg, 2010 in having reddish-brown pronotum, black scutellum and generally black elytra. From these two species, *Gonioctenaklapperichi* sp. nov. can be distinguished by long oval aedeagus (apical 1/3 of aedeagus very slender in *G.scutellaris* and apical 1/3 distinctly narrowed to blunt apex in *G.melanota*).

#### Etymology

The new species is named after the German entomologist, Johann Friedrich Klapperich, who collected the type specimen.

#### Distribution

China: Fujian Province (Guadun).

### Gonioctena (Gonioctena) oberthueri sp. nov.

0109C7E0-B7A4-57C8-B888-85F8F1DB6C38

urn:lsid:zoobank.org:act:6B65B7C4-9343-42D6-A21F-DB59CEA4BCC7

#### Materials

- **Type status:** Holotype. **Occurrence:** individualCount: 1; sex: male; lifeStage: adult; occurrenceID: 4F963831-3A15-54B2-A2DB-16572D62B53D; **Taxon:** scientificName: Gonioctenaoberthueri; kingdom: Animalia; phylum: Arthropoda; class: Insecta; order: Coleoptera; family: Chrysomelidae; genus: Gonioctena; subgenus: Gonioctena; specificEpithet: oberthueri; taxonRank: species; **Location:** higherGeography: China; stateProvince: Sichuan; county: Kangding; **Event:** eventDate: 1896; **Record Level:** institutionCode: MNHN; basisOfRecord: PreservedSpecimen
- **Type status:** Paratype. **Occurrence:** individualCount: 2; sex: female; lifeStage: adult; occurrenceID: 15BF1CDA-735F-5AC3-BADC-B4D96E2C54A3; **Taxon:** scientificName: Gonioctenaoberthueri; kingdom: Animalia; phylum: Arthropoda; class: Insecta; order: Coleoptera; family: Chrysomelidae; genus: Gonioctena; subgenus: Gonioctena; specificEpithet: oberthueri; taxonRank: species; **Location:** higherGeography: China; stateProvince: Sichuan; county: Kangding; **Event:** eventDate: 1896; **Record Level:** institutionCode: MNHN; basisOfRecord: PreservedSpecimen
- **Type status:** Paratype. **Occurrence:** individualCount: 1; sex: female; lifeStage: adult; occurrenceID: 618AB867-B3C8-5CB0-A06E-DC43B5246674; **Taxon:** scientificName: Gonioctenaoberthueri; kingdom: Animalia; phylum: Arthropoda; class: Insecta; order: Coleoptera; family: Chrysomelidae; genus: Gonioctena; subgenus: Gonioctena; specificEpithet: oberthueri; taxonRank: species; **Location:** higherGeography: China; stateProvince: Sichuan; county: Kangding; **Event:** eventDate: 1895; **Record Level:** institutionCode: MNHN; basisOfRecord: PreservedSpecimen

#### Description

Measurements in mm (n = 4): length of body: 6.10–6.80 (mean 6.55); width of body: 3.70–4.10 (mean 3.93); height of body: 2.55–2.90 (mean 2.78); width of head: 1.72–1.80 (mean 1.77); interocular distance: 1.27–1.35 (mean 1.32); width of apex of pronotum: 2.07–2.12 (mean 2.09); width of base of pronotum: 3.15–3.37 (mean 3.30); maximum width of pronotum: 3.17–3.37 (mean 3.30); length of pronotum along mid-line: 1.52–1.62 (mean 1.56); length of elytra along suture: 4.50–5.10 (mean 4.90).

**Figure 3.**
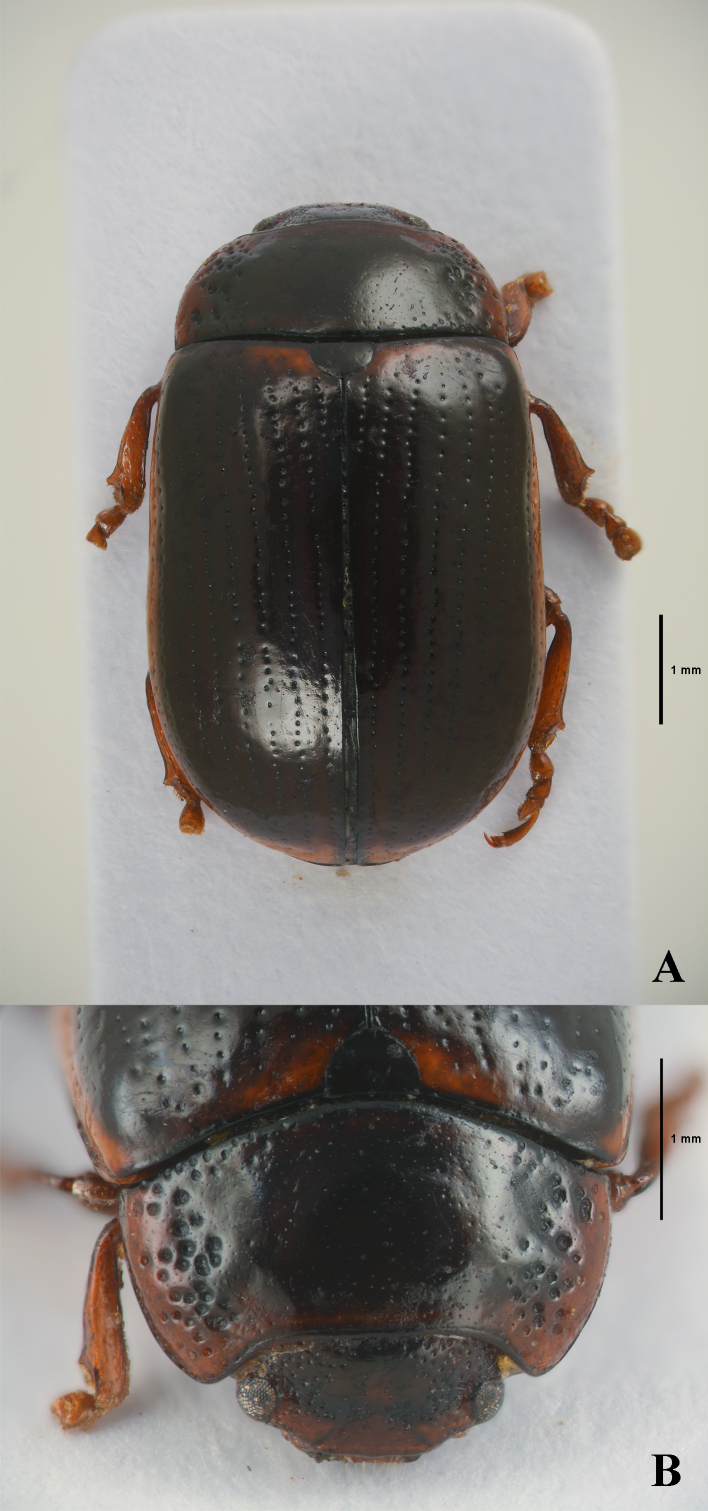
*Gonioctenaoberthueri* sp. nov., holotype. **A** Habitus, dorsal view; **B** Head and pronotum, dorsal view.

**Figure 4.**
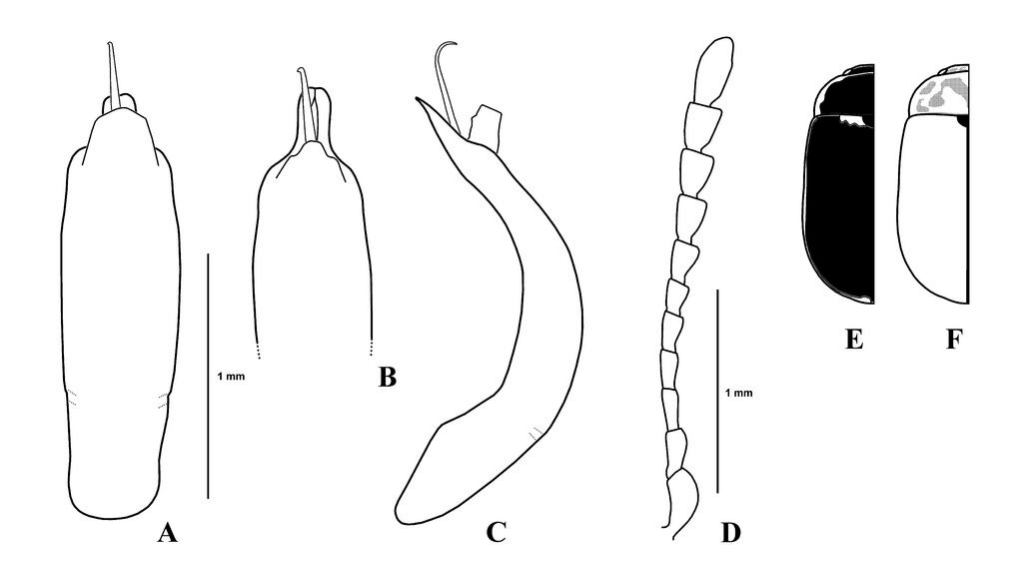
*Gonioctenaoberthueri* sp. nov. **A** Male genitalia, dorsal view; **B** Ditto, apico-dorsal view; **C** Ditto, lateral view; **D** Antenna, female; **E** Dorsal colouration, holotype **F** Ditto, paratypes.

Body (Fig. 3) oblong oval and moderately convex. Head black, with large black markings. Mandibles reddish-brown, apex black. Maxillary palps reddish-brown. Antennae of holotype (male) lost; in female antennomeres I–VII yellowish-brown, VII darkened, VIII–XI black. Pronotum and elytra of holotype (Fig. 4E) mostly black; in paratypes (Fig. 4F) pronotum reddish-brown with obscure markings and elytra reddish-brown with elytral suture and inner margin of epipleura black. Scutellum black to blackish-brown. Venter black to blackish-brown, with prosternum, hypomera and apical margin of last abdominal ventrite reddish-brown. Legs entirely reddish-brown.

Head. Vertex weakly convex, covered with sparse punctures, becoming coarser and denser towards sides. Frontal suture V-shaped, coronal suture rather long and weak. Frons flat, strongly depressed at anterior margin, covered with dense punctures. Clypeus narrow and trapezoidal. Anterior margin of labrum almost straight. Mandibles with two sharp apical teeth and large excavation for apical maxillary palpomere on outer side. Maxillary palps four-segmented, with apical palpomere distinctly widened, truncate apically in male; slightly widened in female. Antennae of female (Fig. 4D) reaching elytral humeri; antennomere I robust; II longer than III; III longer than IV; VII–XI each distinctly longer than wide; XI longest, about 2.17 times as long as wide.

Pronotum. Lateral sides widest at or near base, roundly moderately narrowed anteriorly, anterior angles strongly produced. Anterior and lateral margins bordered, lateral margins barely visible in dorsal view. Trichobothria present on posterior angles. Disc covered with very sparse punctures; lateral sides covered with much coarser and denser punctures, partially confluent; interspaces covered with fine and sparse punctures. Scutellum distinctly wider than long, narrowed posteriorly.

Elytra. Lateral sides slightly widened posteriorly, widest beyond middle, thence roundly narrowed posteriorly. Humeral calli well developed. Disc covered with eleven regular rows of large punctures, including a short scutellar row; interspaces covered with fine and sparse punctures. Epipleura wholly visible in lateral view. Hind wings well developed.

Venter. Hypomera weakly rugose, with dense punctures on anterior side. Prosternum covered with coarse and dense punctures bearing long setae; prosternal process enlarged apically, bordered laterally, with moderately dense punctures. Metaventrite covered with small and sparse punctures in median region, large and dense punctures in lateral region. Abdominal ventrites covered with moderately dense punctures bearing short setae.

Legs. Moderately robust. Tibiae widened apically, with a blunt tooth-like projection. Fore legs with tarsomere I slightly narrower than III. Tarsal claws appendiculate.

Genitalia (Fig. 4A–C). Aedeagus in dorsal view subparallel-sided in middle, with apical process rather short; in lateral view strongly curved, with apical process tapered to pointed apex with long flagellum. Spermatheca absent.

Sexual dimorphism. Apical palpomere of maxillary palps of male is comparatively wider than that of female. The sexual dimorphism of antennae could not be compared due to the damaged antennae of male specimen.

#### Diagnosis

This new species can be easily distinguished from other *Gonioctena* species by the unique colouration, rather elongate antennomeres VII–XI and male genital shape.

#### Etymology

The new species is named after the French entomologist René Oberthür, who had obtained type specimens from a missionary stationed in Kangding.

#### Distribution

China: Sichuan Province (Kangding).

#### Biology

**Figure 5.**
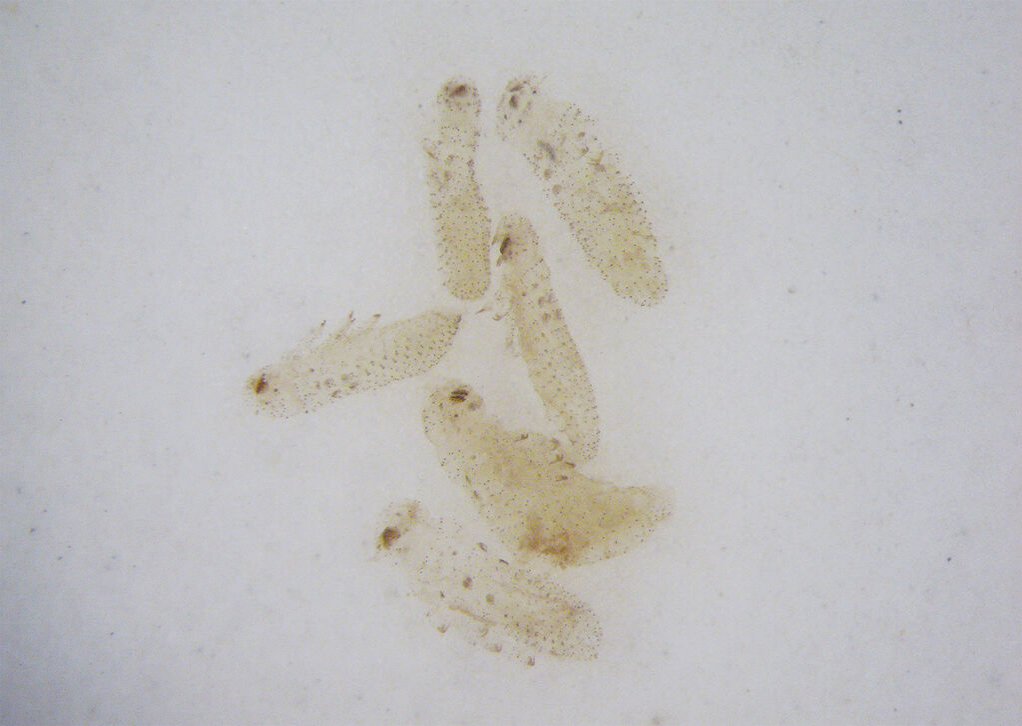
Larvae dissected out from a female of *Gonioctenaoberthueri* sp. nov.

Six larvae were found in the abdomen of one female during the dissection (Fig. 5). Most of species of the subgenus Gonioctena are known to be ovoviviparous (Cho 2022) and this species is newly reported as an ovoviviparous species.

## Supplementary Material

XML Treatment for Gonioctena (Brachyphytodecta) klapperichi

XML Treatment for Gonioctena (Gonioctena) oberthueri
